# Supplementary material for: Biofoundry-Scale DNA Assembly Validation Using Cost-Effective High-Throughput Long-Read Sequencing
Source: ACS Synth Biol. 2024 Feb 8;13(2):683–6. doi: 10.1021/acssynbio.3c00589 (PMC10877595; doi:10.1021/acssynbio.3c00589)
Supplement: Supplementary file 1 — sb3c00589_si_001.pdf [file sb3c00589_si_001.pdf]

SUPPORTING INFORMATION

Biofoundry-scale DNA assembly validation using cost-effective high-throughput long-read sequencing

Peter Vegh[1], Sophie Donovan[1], Susan Rosser[1], Giovanni Stracquadanio[1], Rennos Fragkoudis[1,2]

Affiliation: [1]Edinburgh Genome Foundry, School of Biological Sciences, University of Edinburgh, Edinburgh, EH9 3BF, United Kingdom

[2]Department of Biochemistry and Biotechnology, University of Thessaly, 41500 Larissa, Greece

Contents:

Supporting Information S1: an example ‘analysis’ report generated by the Sequeduct pipeline.

Supporting Information S2: an example ‘review’ report generated by the Sequeduct pipeline.

The PDF reports and corresponding data can also be found at the demonstration repository of the pipeline: https://github.com/Edinburgh-Genome-Foundry/Sequeduct_demo/

The reports were created by the Ediacara Python package, developed as part of the Sequeduct pipeline: https://github.com/Edinburgh-Genome-Foundry/Ediacara

Supporting Information S1

An example ‘analysis’ report generated by the Sequeduct pipeline. The report can be found on the following pages.

Sequencing analysis report

This document reports the sequencing results of the **EGF demo** project. There are **2** barcodes in the analysis. Each chapter details the results of one barcode, and within a chapter each section reports on one plasmid. Please see the Appendix on the last page for an explanation of the report.

There are **1623** filtered reads in the analysed barcodes of this sequencing run.

Generated on 2023-08-24 by Ediacara (version 0.2.2)

Barcode: barcode01

Results for the **1** construct(s) in the pool: **1** / **0** / **0** / **0** (pass / warning / fail / low coverage).

Name	Result	Length [bp]	FASTQ reads	Coverage [x]
**EGF2_2**	PASS	7604	792	557

Histogram of the **792** FASTQ reads:

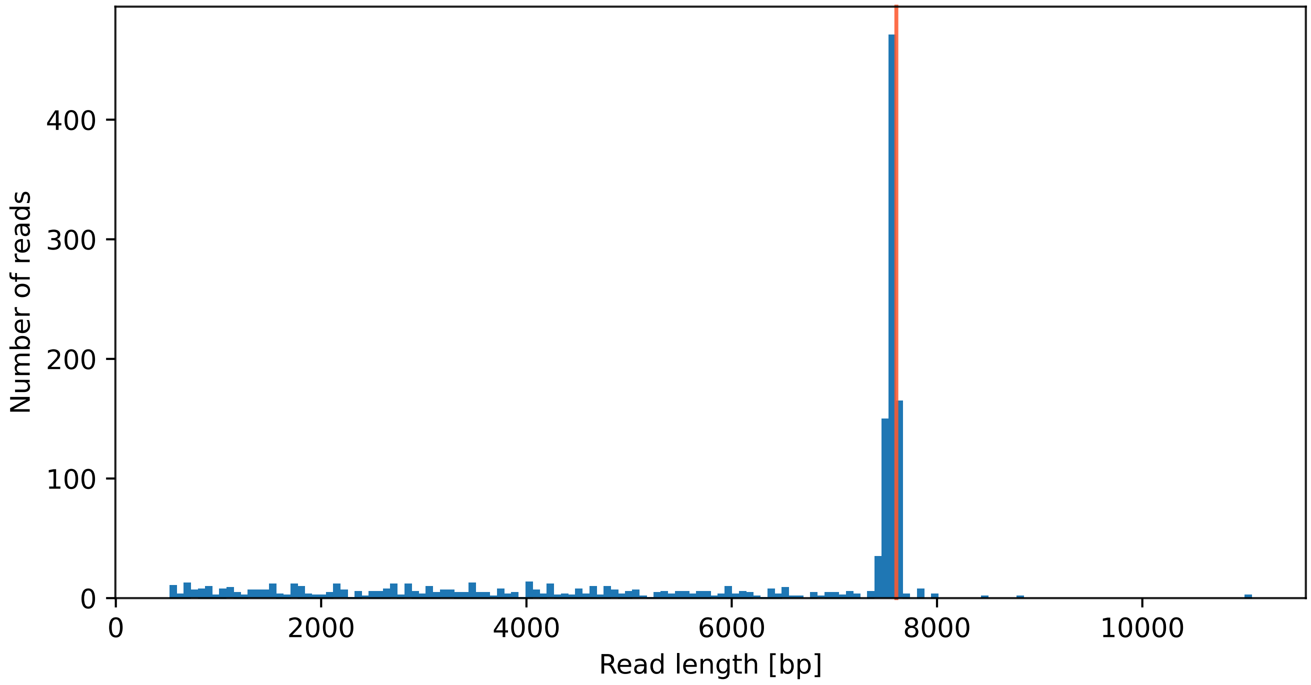

The vertical red lines show the expected construct lengths.

Generated on 2023-08-24 by Ediacara (version 0.2.2)

☑ EGF2_2

Result: PASS

Length of reference: **7604** bp.

Coverage plot

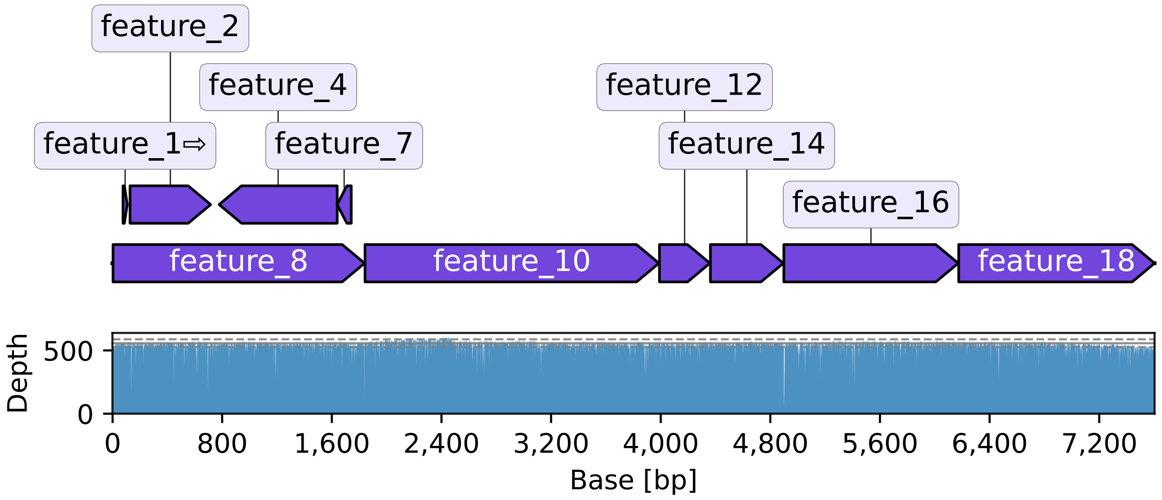

Positions with zero coverage: -

Positions with low coverage (<50%): 137, 448, 695, 1190, 1837, 3891, 4899-4902, 5164, 5413, 6465

Cumulative plot of longest unaligned interval in each read (2% above cutoff. mode: 11 bp):

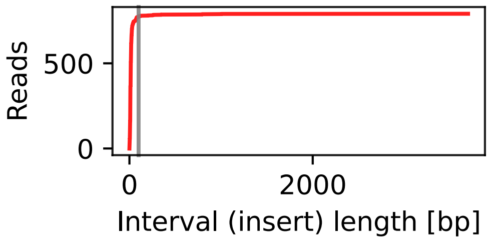

Comparison with reference

LOC	REF	ALT	TYPE	DP	RO	AO	T
**447**	CGGGGGGTTC	CGGGGGTTC	del	154	43	72	0
**1836**	TCA	TA	del	131	0	126	1
**4898**	TCCCCCCCCCCTAAC...	TCCCCCCCTAACG	del	91	3	22	0

Generated on 2023-08-24 by Ediacara (version 0.2.2)

EGF2_2 reference vs consensus of reads:

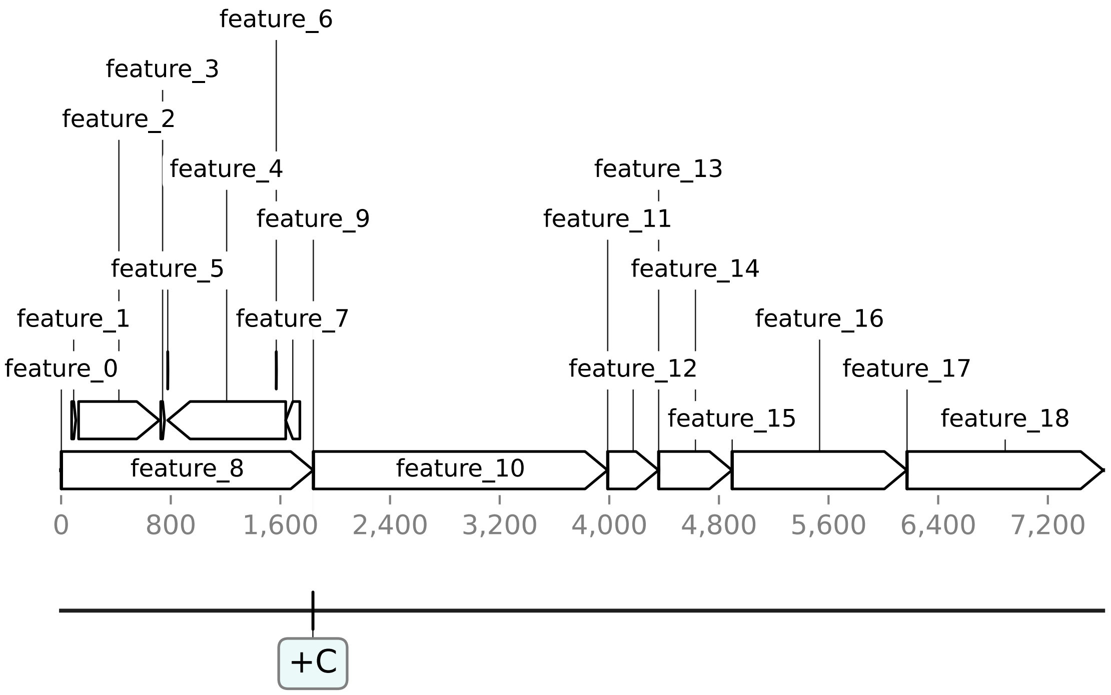

Barcode: barcode12

Results for the **1** construct(s) in the pool: 0 / 0 / 1 / 0 (pass / warning / fail / low coverage).

Name	Result	Length [bp]	FASTQ reads	Coverage [x]
**EGF2_13**	FAIL	8939	831	592

Histogram of the **831** FASTQ reads:

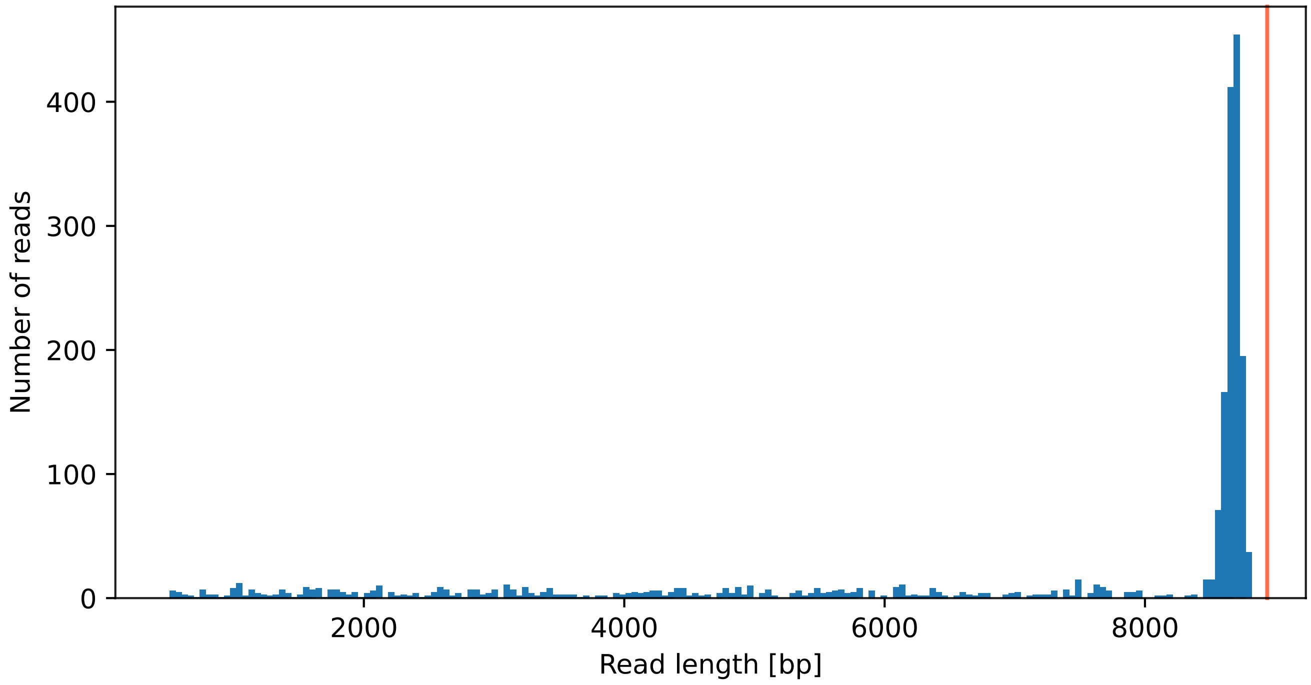

The vertical red lines show the expected construct lengths.

Generated on 2023-08-24 by Ediacara (version 0.2.2)

Generated on 2023-08-24 by Ediacara (version 0.2.2)

☒ EGF2_13

Result: FAIL

Length of reference: **8939** bp.

Coverage plot

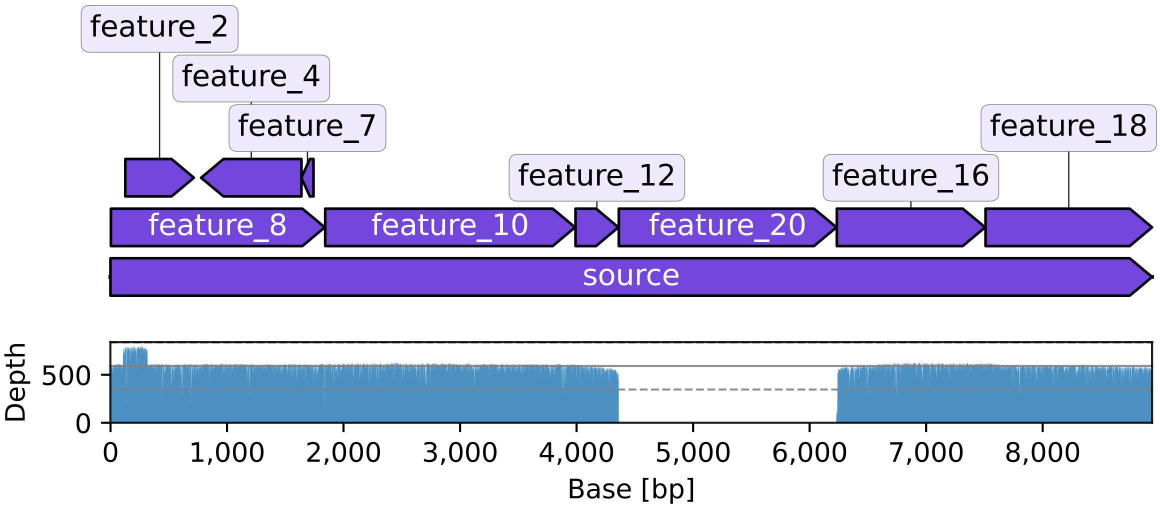

Positions with zero coverage: 4376-6227

Positions with low coverage (<50%): 137, 448, 695, 1837, 3882, 4362-6237, 6499, 6748, 7800

Cumulative plot of longest unaligned interval in each read (81% above cutoff. mode: 1656 bp):

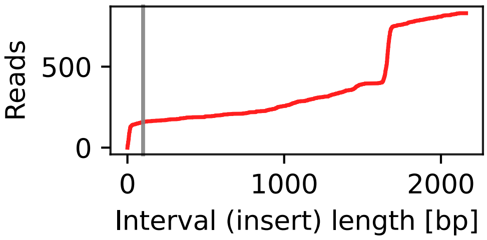

Comparison with reference

LOC	REF	ALT	TYPE	DP	RO	AO	T
**1836**	TCA	TA	del	140	1	136	1
**6233**	TCCCCCCCCCCTAAC...	TCCCCCCCCTAACG	del	31	3	15	0

EGF2_13 reference vs consensus of reads:

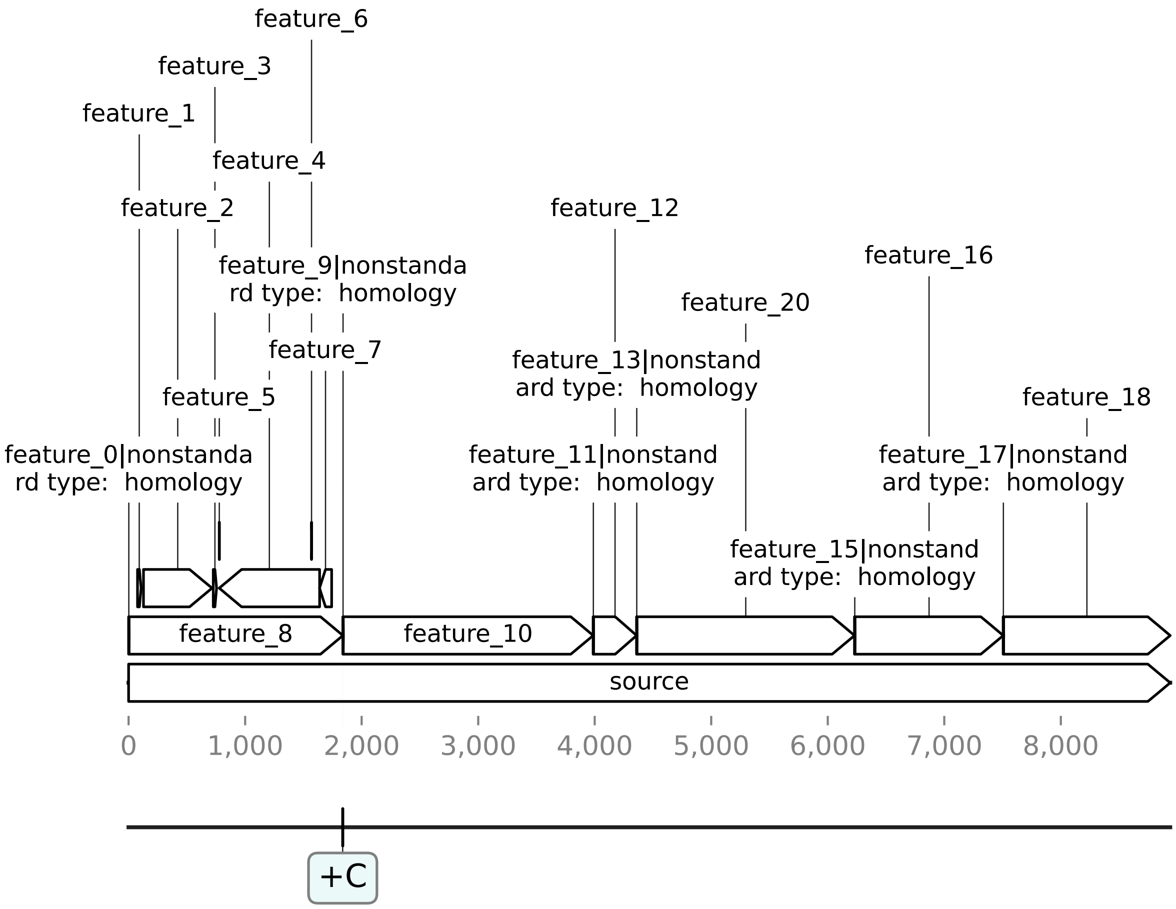

Generated on 2023-08-24 by Ediacara (version 0.2.2)

Generated on 2023-08-24 by Ediacara (version 0.2.2)

Appendix

Each barcode chapter consists of a summary and analysis sections.

Summary page

The first page summarises the results and other details for each plasmid construct.

Analysis pages

Each plasmid construct is analysed separately. The result is summarised with a symbol:
☑ / : correct construct, based on no detection of errors
⚠ / : warning signs are present, review the results and make a decision
☒ / : errors were detected in the construct
⍰ / : sequencing problems, insufficient reads

Depending on settings and input data, some of the described plots may not be in the report.

Coverage

The coverage plot displays the number of reads aligning to each base of the reference, in blue. This is useful for detecting deletions. The grey line shows the median. The dashed lines show the ±stdev. Positions with low coverage are also listed, if there are any. Low coverage = below the coverage threshold.

Comparison with reference

If a consensus (or *de novo* assembly) sequence is created from the reads, then it is compared with the reference. If the lengths differ more than 5%, then an error is displayed here, otherwise a GeneBlocks plot is made. In this plot, the reference is displayed, and annotated with changes compared to the consensus obtained from the reads (unless a note tells otherwise). Fo example, +G means that the reference file has an extra G compared to the DNA sample, in other words, the DNA has a G deletion. A VCF (variant call format) table is also displayed. Both the reported and the VCF table positions are zero-based, but the VCF table reports the position of the variation, rather than the nucleotide. Note that the reported depths (DP) may be lower than the ones in the coverage plot, depending on how the variant call was performed. The columns of the table are:

- LOC: 0-based position index (where the first nucleotide has index 0)
- REF: Reference sequence
- ALT: Alternative sequence
- TYPE: The type of allele (either snp, mnp, ins, del or complex)
- DP: Total read depth at the locus
- RO: Reference allele observation count
- AO: Alternate allele observations

An additional column T is provided to mark entries (1) that are deemed true mutations. Inconclusive mutations at repeats (homopolymers) were shown to be systematic sequencing errors, and can be ignored.

Ediacara

The report was generated by Ediacara, a software published by the Edinburgh Genome Foundry (EGF). Ediacara is part of the EGF Codons engineering biology software suite for DNA design, manufacturing and validation.

Generated on 2023-08-24 by Ediacara (version 0.2.2)

Supporting Information S2

An example ‘review’ report generated by the Sequeduct pipeline. The report can be found on the following pages.

Edinburgh Genome Foundry

Assembly analysis report

This document reports the sequencing results of the **EGF demo review** project. Each chapter details the results of a plasmid (assembly). Please see the Appendix on the last page for an explanation of the report.

Generated on 2023-08-24 by Ediacara (version 0.2.2)

EGF2_2

Note: the assembly is in reverse complement, compared to the reference.

Plot of the aligning parts:

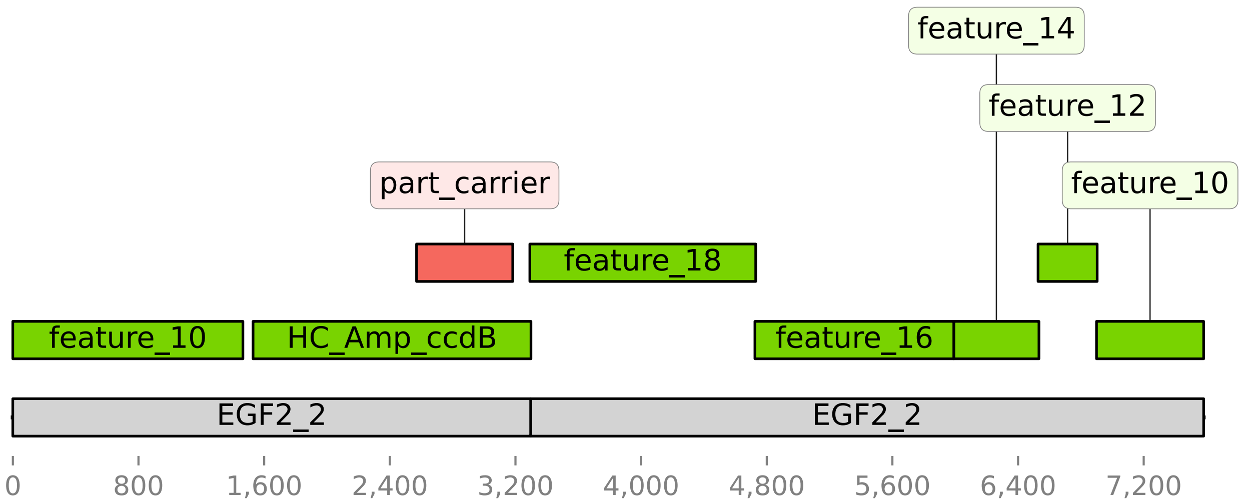

Alignment table:

Name	Length	Start	End	Strand	T Start	T End	Matches	Size	Quality
**EGF2_2**	7604	3307	7604	-	3297	7582	4285	4297	60
**EGF2_2**	7604	0	3307	-	0	3297	3297	3307	60
**HC_Amp_ccdB**	2721	947	2721	-	1530	3299	1769	1774	60
**feature_10**	2166	6	1475	-	0	1465	1465	1469	60
**feature_10**	2166	1475	2159	-	6900	7582	682	684	60
**feature_12**	390	7	383	-	6528	6904	376	376	60
**feature_14**	554	6	547	-	5992	6533	541	541	60
**feature_16**	1294	15	1287	-	4725	5992	1267	1272	60
**feature_18**	1451	6	1445	-	3292	4730	1438	1439	60
**part_carrier**	2329	235	852	+	2571	3183	611	617	60

Generated on 2023-08-24 by Ediacara (version 0.2.2)

EGF2_13

Note: the assembly is in reverse complement, compared to the reference.

Plot of the aligning parts:

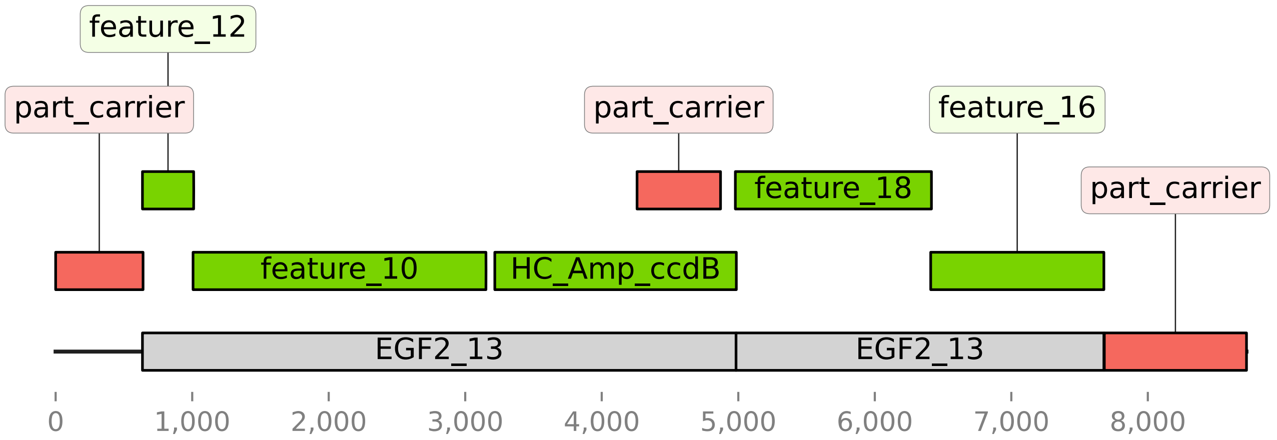

Alignment table:

Name	Length	Start	End	Strand	T Start	T End	Matches	Size	Quality
**EGF2_13**	8939	0	4362	-	636	4984	4348	4362	60
**EGF2_13**	8939	6238	8939	-	4984	7678	2694	2701	60
**HC_Amp_ccdB**	2721	947	2721	-	3217	4986	1769	1774	60
**feature_10**	2166	6	2159	-	1007	3152	2145	2153	60
**feature_12**	390	7	382	-	636	1011	375	375	60
**feature_16**	1294	15	1287	-	6410	7678	1268	1272	60
**feature_18**	1451	6	1445	-	4979	6415	1436	1439	60
**part_carrier**	2329	1287	2329	-	7682	8723	1041	1042	60
**part_carrier**	2329	646	1287	-	0	640	640	641	60
**part_carrier**	2329	235	852	+	4259	4870	610	617	60

Generated on 2023-08-24 by Ediacara (version 0.2.2)

Appendix

Each chapter describes results on a plasmid sequence (*de novo* assembly). The provided (part) sequences were aligned against the sequence assembled from the reads. The plot shows alignment regions as annotations. If there are unannotated segments, then none of the parts aligned there. If an assembly plan is provided, then annotations are coloured based on whether they are expected in the plasmid: green: part is expected in the construct; red: part shouldn't be in the construct. Grey is the reference expected sequence for the plasmid. The alignments are provided in a PAF (pairwise mapping format) table. The columns of the table are:

- Name: Query sequence name
- Length: Query sequence length
- Start: Query start coordinate
- End: Query end coordinate
- Strand: `+` if query and target on the same strand; `–` if opposite
- T Start: Target start coordinate on the original strand
- T End: Target end coordinate on the original strand
- Matches: Number of matching bases in the mapping (includes mismatches but not indels)
- Size: Number bases, including gaps (indels), in the mapping
- Quality: Mapping quality (0–255 with 255 for missing)
